# Neurotherapeutic Potential of *Cervus elaphus* Sibericus on Axon Regeneration and Growth Cone Reformation after H_2_O_2_-Induced Injury in Rat Primary Cortical Neurons

**DOI:** 10.3390/biology10090833

**Published:** 2021-08-26

**Authors:** Jin Young Hong, Junseon Lee, Hyunseong Kim, Wan-Jin Jeon, Changhwan Yeo, Bo Ram Choi, Jee Eun Yoon, Ji Yun Shin, Jeom-Yong Kim, In-Hyuk Ha

**Affiliations:** 1 Jaseng Spine and Joint Research Institute, Jaseng Medical Foundation, Seoul 135-896, Korea; vrt23@jaseng.org (J.Y.H.); excikind@jaseng.org (J.L.); biology@jaseng.org (H.K.); cool2305@jaseng.org (W.-J.J.); duelf2@jaseng.org (C.Y.); 2 Jaseng Bio Science Research Institute, Jaseng Bio. Co., Ltd., Seoul 061010, Korea; bohosun@jsd1.co.kr (B.R.C.); jee0527@jsd1.co.kr (J.E.Y.); jasengbio@jsd1.co.kr (J.Y.S.)

**Keywords:** axon regeneration, filopodia, growth cone, retraction bulb, *Cervus elaphus* sibericus, cortical neurons, hydrogen peroxide, brain-derived neurotrophic factor, nerve growth factor, regeneration-associated genes

## Abstract

**Simple Summary:**

Oxidative stress plays a crucial role in nerve injury-induced changes, including the cellular redox state, greater disease severity, and reduced function. Most studies have demonstrated that the modulation of oxidative damage to neurons leads to improved survival and functional recovery. Deer antlers are considered a potent natural source of antioxidants. Thus, we examined the neurotherapeutic effect of *Cervus elaphus* sibericus in the axon regeneration and growth cone reformation of cortical neurons. The effect of three doses of *C. elaphus* sibericus on cortical neurons was determined in a hydrogen peroxide-induced injury model using immunocytochemistry, flow-cytometry, and real-time PCR. We found that *C. elaphus* sibericus effectively inhibited neuronal death following hydrogen peroxide-induced injury by significantly inhibiting oxidative stress and consequently promoting neuron repair and axon regeneration in cortical neurons.

**Abstract:**

*Cervus elaphus* sibericus (CES), commonly known as deer antler, has been used as a medicinal herb because of its various pharmacological activities, including its anti-infective, anti-arthritic, anti-allergic, and anti-oxidative properties. However, the precise mechanisms by which CES functions as a potent anti-oxidative agent remain unknown; particularly, the effects of CES on cortical neurons and its neurobiological mechanism have not been examined. We used primary cortical neurons from the embryonic rat cerebral cortex and hydrogen peroxide to induce oxidative stress and damage in neurons. After post-treatment of CES at three concentrations (10, 50, and 200 µg/mL), the influence of CES on the neurobiological mechanism was assessed by immunocytochemistry, flow cytometry, and real-time PCR. CES effectively prevented neuronal death caused by hydrogen peroxide-induced damage by regulating oxidative signaling. In addition, CES significantly induced the expression of brain-derived neurotrophic factor and neurotrophin nerve growth factor, as well as regeneration-associated genes. We also observed newly processing elongated axons after CES treatment under oxidative conditions. In addition, filopodia tips generally do not form a retraction bulb, called swollen endings. Thus, CES shows therapeutic potential for treating neurological diseases by stimulating neuron repair and regeneration.

## 1. Introduction

Increased oxidative stress is a general pathophysiological property of many neurological disorders, such as Alzheimer’s disease, Parkinson’s diseases, and spinal cord and brain trauma [1,2,3]. Once a nerve is damaged, reactive oxygen species (ROS) generation dramatically increases and contributes to cellular dysfunction, neuronal cell death, and tissue damage [4]. In particular, high levels of ROS appear to cause environmental oxidate stresses concomitant with focal axonal degeneration, neuropathic pain, and locomotor dysfunction [5]. In fact, oxidative stress is closely associated with various pathological conditions, such as aging, diabetes, arthritis, cancer, atherosclerosis, and neurodegenerative and cardiovascular diseases [6,7]. Therefore, approaches for regulating oxidative stress may improve biological function and recovery. Previous studies revealed that natural antioxidants can effectively improve neuronal viability and axonal regeneration, and attenuate elevated ROS levels [8,9]. *Cervus elaphus* sibericus (CES), commonly known as deer antler, is an herbal medicine that has long been regarded as a powerful natural substance with diverse pharmacological activities, including anti-infective, anti-arthritic, anti-allergic, and anti-oxidative effects [10,11,12,13,14,15]. In particular, regarding its antioxidant potential, CES contains numerous biologically active substances, such as uronic acid, sulfated glycosaminoglycan, sialic acid, uridine, uracil, and hypoxanthine, and possesses a high antioxidant potential [16]. Thus, CES has been shown to act as an ROS scavenger. CES increases the survival and proliferation of SH-SY5Y, HT-22, and BV2 cells in vitro by protecting against oxidative stress-induced damage [17,18,19].

Furthermore, CES has shown antioxidant effects in murine models of obesity, rheumatoid arthritis, fatty liver, and nephrotoxicity [11,20,21,22], and previous studies, particularly those focusing on neurological disease, have reported that the methanol extracts of velvet antler ameliorate Parkinson’s disease by inhibiting oxidative stress and neuroinflammation [18]. Although these results were demonstrated in neuronal-like cell lines and various disease models, neurotherapeutic effects have not been directly observed in mature neurons. Several neuronal cell lines, such as P19, PC12, and SH-SY5Y cells, were generated from carcinoma or neuroblastoma and based on the characteristics of stem cells [23]. However, fully differentiated central nervous system neurons cannot divide to reform new neuronal populations after injury because they lack a proliferation ability [24].

Therefore, the therapeutic effects and mechanisms occurring during neural repair and axon regeneration should be demonstrated in cells that share the morphological and biochemical characteristics of mature neurons. In the present study, mature cortical neurons were used to examine the therapeutic effects of CES. We applied three doses of CES to an in vitro hydrogen peroxide (H_2_O_2_)-treated model of cortical neurons and examined neuronal viability and axon regeneration. We focused on changes in the axonal growth cone following neuron injury. Growth cones regulate the extension of a regenerating neurite and act as guidance cues during development [25]. After central nerve injury, injured central nervous system axons form axonal bulbs with a typical ball shape in the tip of their proximal stumps along the axons. Axonal bulbs induce axonal swelling and degeneration, and consequently regeneration failure [26]. Thus, growth cone reformation at the growing tip of axons is a crucial step in subsequent and successful axonal regeneration. We demonstrated the in vitro neurotherapeutic effect of CES in H_2_O_2_-induced neuronal injury in cortical neurons and the morphological alterations of the growth cone, suggesting that CES has neurotherapeutic potency for preventing and treating neurological disorders.

## 2. Materials and Methods

### 2.1. In Vitro Culture of Cortical Neurons

Primary cortical neurons were harvested from Sprague-Dawley rats at the age of 17 embryonic days. All experiments involving animals were conducted according to the Use Committee guidelines (JSR-2021-07-003-A) with Jaseng Animal Care. Details of the cortical culture procedures have been previously described [27]. Briefly, cell culture plates were coated with poly-D-lysine (Gibco BRL, Grand Island, NY, USA) and laminin (Gibco BRL). The brains were separated from the embryos, placed in the Hank’s balanced salt solution (Gibco BRL), and carefully divided into cerebral hemispheres. The meninges and hippocampus were manually removed to obtain a pure cortical neuron. Enzymatic digestion was performed using a Neural Tissue Dissociation kit (Miltenyi Biotec, Bergisch Gladbach, NRW, Germany) and GentleMACS^TM^ Octo Dissociator (Miltenyi Biotec) to prepare single cells. After 20 min at 37 °C, the cells were washed twice with 2 mL of Hank’s balanced salt solution and centrifuged for 3 min at 2000 rpm to obtain the cell pellet. The supernatant was carefully removed, and the cell pellet was gently resuspended in 1 mL of neurobasal medium containing 2% B27 (Gibco BRL), 1% Gluta-MAX (Gibco BRL), 1% penicillin/streptomycin (Gibco BRL), and recombinant brain-derived neurotrophic factor (BDNF; Peprotech, Cranbury, NJ, USA). The cells were seeded onto coated 12-mm glass coverslips (Paul Marienfeld GmbH and Co., Lauda-Königshofen, Germany) in 24-well plates at 2 × 10^4^ cells/450 µL for immunocytochemistry, 6-well plates at 2 × 10^6^ cells/1.8 mL for fluorescence-activated cell sorting analysis, 60-mm^2^ dishes at 4 × 10^6^ cells/2.7 mL for real-time PCR, and 96-well plates at 2 × 10^4^ cells/90 µL for cell viability assessment.

### 2.2. Preparation of CES Extract

The CES extract was prepared as previously described [28]. All parts of the Russian dear antler were heated for 24 h at 103 °C in sterile water and filtered through filter paper (HA-030, Hyundai Micro, Seoul, Korea). This mixture was concentrated using a rotary evaporator (Eyela, Koishikawa, Tokyo, Japan) and then lyophilized in a freeze dryer (Ilshin BioBase, Gyeonggi-do, Korea) to obtain the CES dry extract. The CES dry extract was diluted to the 100 mg/mL in phosphate-buffered saline (PBS) and stored at −20 °C until use.

### 2.3. H_2_O_2_-Induced Oxidative Injury and CES Treatment

**Scheme 1:**
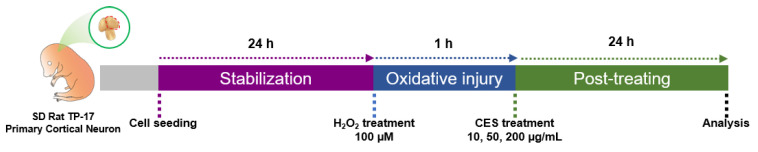
Schematic timeline of the experimental procedures in an H_2_O_2_ condition.

H_2_O_2_, 30% (*w*/*w*), used to prepare the stock solutions, was obtained from Sigma (Sigma Aldrich, St. Louis, MO, USA). The stock solution was freshly prepared at 100 mM for each experiment, and oxidative injury was induced by adding 500/~1 of 100 mM H_2_O_2_ solution to the culture medium. After 1 h, the culture medium was discarded and replaced with new medium containing 10, 50, or 200 µg/mL CES extract, and then incubated in 5% CO_2_ and 37 °C for 24 h. The experimental timeline is described in Scheme 1.

### 2.4. Laceration Injury

**Scheme 2:**
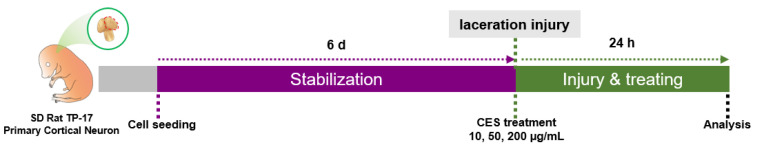
Schematic timeline of the experimental procedures in the laceration injury.

Laceration injury was performed based on a previous method described by Stupack (2020) [29]. Briefly, cortical neurons were cultured in a neuronal culture medium on coated 12-mm glass coverslips in 24-well culture plates until day 6 in vitro. Neurites were then mechanically wounded by dragging a 10-µL pipette tip centrally across the coverslip, followed by treatment with CES at 10, 50, or 200 µg/mL and fixation of the cells after 24 h. The experimental timeline is described in Scheme 2.

### 2.5. Neuronal Viability Assays

Neuronal viability was evaluated using a Cell Counting Kit-8 assay (CCK-8; Dojindo, Kumamoto, Japan) and with a live/dead cell imaging kit (Thermo Fisher Scientific, Waltham, MA, USA). First, the cells were added to a 96-well plate for the CCK assay and treated with various concentrations of CESs (1, 10, 50, 200, and 500 µg/mL) with or without H_2_O_2_ exposure. After incubation for 24 h, 10 μL of CCK-8 solution was added to each well. After 4 h, absorbance was measured at 450 nm using a microplate reader (Epoch, BioTek, Winooski, VT, USA). Cell viability was calculated as the percentage of surviving neuron cells relative to the value of the blank group.

A live/dead cell imaging kit (Invitrogen, Grand island, NY, USA) was used to visualize and verify neural viability. The dyeing solution contained two probes: calcein AM, which marks living cells as green, and ethidium homodimer-1, which marks dead cells as red. The cells were prepared on each coverslip in a 24-well plate and treated with 10, 50, or 200 µg/mL of CES with H_2_O_2_ exposure. After the cells were incubated for 24 h, the culture medium was replaced with a cortical neuron medium containing a dye solution and incubated at 37 °C for 15 min. After dyeing, the samples were rinsed in PBS and mounted with a fluorescence mounting medium (Dako Cytomation, Carpinteria, CA, USA). All of the images were randomly captured at 100× magnification with a confocal microscope (Eclipse C2 Plus, Nikon, Minato City, Tokyo, Japan). Live/dead cells were quantified by counting the number of green or red-stained cells using ImageJ software (1.37 v, National Institutes of Health, Bethesda, MD, USA).

### 2.6. Immunocytochemistry

Immunocytochemistry was performed to investigate the antioxidative and neurotherapeutic effects of CES. Cells were fixed with 4% paraformaldehyde for 10 min at room temperature, washed three times for 5 min each with PBS, and then permeabilized with 0.2% Triton X-100/PBS for 5 min. Next, the cells were washed twice with PBS for 5 min each time, and blocked for 1 h in 2% normal goat serum. All of the primary antibodies were diluted in 2% normal goal serum and refrigerated for 16 h as follows: rabbit anti-BDNF (1:200; Abcam, Cambridge, UK), rabbit anti-NGF (1:100; Abcam), rabbit anti-Synapsin1 (1:500; Synaptic Systems, Goettingen. Germany), rabbit or mouse anti-Tuj1 (1:2000; R&D Systems, Minneapolis, MN, USA), rhodamine phalloidin (F-actin; 1:1000; Invitrogen), and mouse anti-iNOS (1:100; R&D systems). After washing the cells three times with PBS for 5 min each, secondary antibodies (FITC-conjugated goat anti-mouse or rabbit IgG, Rhodamine goat anti-mouse or rabbit IgG, Jackson Immuno-Research Labs, West Grove, PA, USA) were diluted to 1:300 in 2% normal goat serum and were incubated at room temperature for 2 h, followed by washing three times with PBS for 5 min each time. The cell nuclei were stained for 10 min with diamond-no-2-phenylindole (DAPI; Tokyo Chemical Industry Co., Tokyo, Japan), washed twice with PBS for 5 min each time, and mounted with a fluorescence mounting medium (Dako Cytomation). Images were captured at the same acquisition settings under 100× or 400× magnification by confocal microscopy (Eclipse C2 Plus) to quantify the fluorescence intensity and were measured by ImageJ software (1.37 v, National Institutes of Health).

### 2.7. Axon and Growth Cone Quantification

Axons were quantified from 400× magnification images for total, mean, and maximal neurite outgrowth using ImageJ (1.53 v, Fiji Distribution, National Institute of Health). In addition, growth cones were quantified using the following three parameters: (1) the maximal diameter of growth cone at the tip of axon (µm), (2) the area of grow cone (µm^2^), and (3) the percentage of axons with retraction bulbs = the number of axons with retraction bulb/total number of axons × 100 (%) with ImageJ software (1.37 v, National Institutes of Health) at 400× magnification [26,30].

### 2.8. Flow Cytometry

Flow cytometric assays were performed to confirm the cell death and ROS production. For cell death analysis, cultured cells were stained with an Annexin V-PE/PI apoptosis detection kit (Abcam). Briefly, the cell pellet was resuspended in 100 µL of 1× binding buffer containing 5 µL each of Annexin V-PE and propidium iodide, and then immediately analyzed by fluorescence-activated cell sorting (Accuri C6 plus flow cytometer, BD Biosciences, Franklin Lakes, NJ, USA). For the ROS analysis, 2′,7′-dichlorofluorescin diacetate (DCFDA, Sigma) was used to detect the intracellular ROS levels. Briefly, we prepared a stock solution at 5 mM by dissolving 9.3 mg DCFDA powder in high-quality 3.8 mL anhydrous dimethyl sulfoxide (DMSO; Sigma) and added 1 mL of 10 µM DCFDA solution to the cell pellet. The mean positive cell values, as determined by flow cytometry, were expressed as percentages relative to the control group.

### 2.9. Real-Time PCR

We examined the effects of CES on axon regeneration and antioxidant in cortical neurons by measuring the expression levels of *Nrf2, BDNF*, *NGF*, *NF200,* and *GAP43* using real-time PCR. The total RNA was isolated using TRIzol reagent (Ambion, Austin, TX, USA). cDNA was synthesized using random hexamer primers and Accupower RT PreMix (Bioneer, Daejeon, Korea). All of the primer pairs were designed using the UCSC Genome Bioinformatics and NCBI databases, and their sequences are listed in Table 1. Real-time PCR was performed using iQ SYBR Green Supermix (Bio-Rad, Hercules, CA, USA) on a CFX Connect Real-Time PCR Detection System (Bio-Rad). All real-time PCR steps were performed in at least triplicate. The expression of the target genes was normalized to GAPDH and is shown as the fold-change relative to the control group.

### 2.10. Statistical Analysis

Statistical analysis was performed using GraphPad Prism software (v8.5, GraphPad, Inc., La Jolla, CA, USA). One-way analysis of variance followed by Tukey’s post hoc test was used for multiple comparisons of all data between the groups. Differences were considered as statistically significant if the *p* value was # *p* < 0.001 vs. the blank group and * *p* < 0.05, ** *p* < 0.01, *** *p* < 0.001, or **** *p* < 0.0001 vs. the control group.

## 3. Results

### 3.1. CES Protect Neurons from H_2_O_2_-Induced Neuronal Death in Cortical Neurons

**Figure 1:**
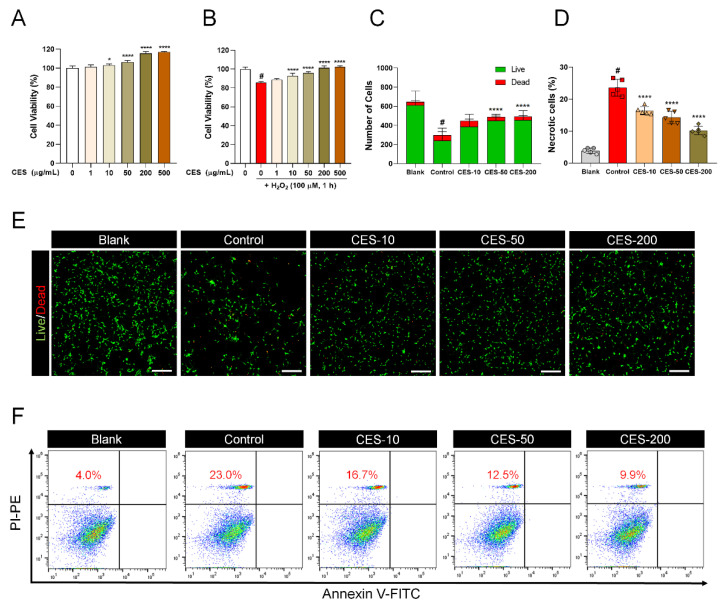
Neuroprotectic effect of CES on H_2_O_2_-induced neurotoxicity in rat primary cortical neurons. (**A**) Cell viability of cortical neurons treated with CES determined only by CCK assay. (**B**) Cell viability of H_2_O_2_-induced cortical neurons treated with CES determined by CCK assay. (**C**) Quantification of cell viability from live/dead images. (**D**) Quantification by flow cytometry with Annexin-V/-FITC/PI positivity. (**E**) Fluorescent images of live/dead assay on H_2_O_2_-induced cortical neurons with various concentrations of CES. White scale bar = 200 µM. (**F**) Representative flow cytometry plots using Annexin-V/-FITC/PI. Data are expressed as the means ± SEM. Significant differences indicated as # *p* < 0.001 vs. blank group, * *p* < 0.05 and **** *p* < 0.0001 vs. control group were analyzed by one-way ANOVA with Tukey’s post hoc test.

First, we investigated the toxicity of CES on primary cortical neurons with or without exposure to H_2_O_2_. The cortical neurons were treated with various doses of CES to determine whether CES was cytotoxic towards cortical neurons. CES were not toxic to cortical neurons at doses of 1–500 µg/mL, with cell viability significantly increased from 10 to 500 µg/mL CES (Figure 1A). We also confirmed the optimal therapeutic dose range of CES in the presence of H_2_O_2_. Neuronal cell viability was significantly decreased after H_2_O_2_ treatment compared with in the blank group; in contrast, CES significantly increased cell viability, starting at a dose of 10 µg/mL, in a dose-dependent manner (Figure 1B). The optimal doses of 10, 50, and 200 µg/mL CES were determined using a CCK assay. A live and dead cell assay was also performed to confirm the neuroprotective effect of these optimal CES doses. The number of living cells (green) was dramatically reduced after H_2_O_2_ treatment, showing a significant difference compared with the blank group. Many green-stained viable cells were observed at all doses of CES, with a significant increase induced by 50 and 200 µg/mL of CES (Figure 1C,E).

Next, we confirmed the ability of CES to suppress neuronal death induced by H_2_O_2_ using flow cytometry with Annexin V/PI staining. PI-positive necrotic cells were detected after H_2_O_2_ treatment, approximately 23% of the cells died due to H_2_O_2_-induced necrotic death (annexin V−/PI+) at 10,000 single cell events collected for analysis gate. In contrast, CES treatment produced a dose-dependent decrease in the rate of PI-positive necrotic cells, reaching a minimum of approximately 10% (Figure 1D,F). Based on these results, CES can not only enhance neuronal cell viability, but also decrease H_2_O_2_-induced oxidative death.

### 3.2. CES Suppresses H_2_O_2_-Promoted iNOS Expression and ROS Production in Cortical Neurons by Activating the Nrf2 Pathway

**Figure 2:**
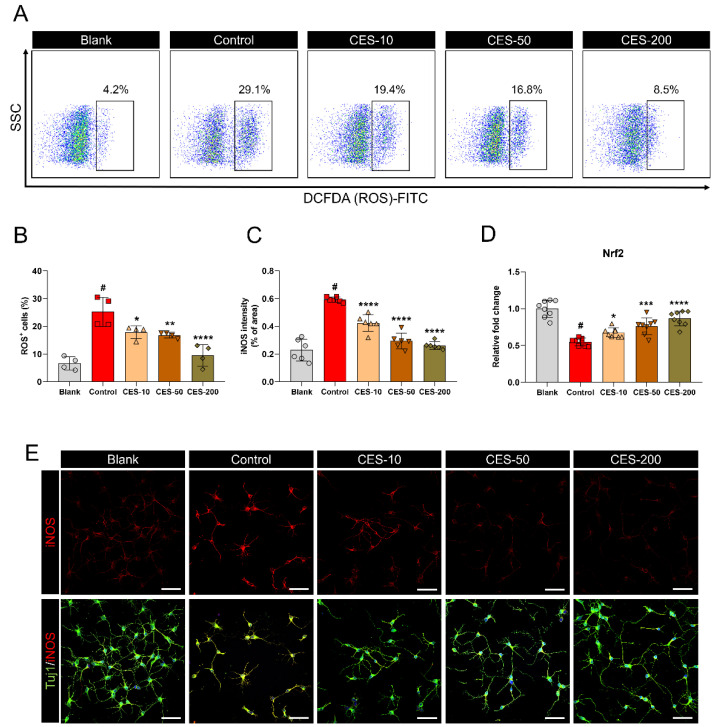
Antioxidative effect of CES on H_2_O_2_-induced oxidative stress in rat primary cortical neurons. (**A**) Representative flow cytometry plots showing DCFDA-ROS. (**B**) Quantification of ROS production measured by flow cytometry with DCFDA positivity. (**C**) Relative fluorescence intensity of iNOS-stained neurons. (**D**) *Nrf2* expression determined by real-time PCR in cortical neurons after H_2_O_2_ and CES application. (**E**) Representative image of the neuronal marker Tuj1 (green) and iNOS (red) double-staining. White scale bar = 50 µM. Data are expressed as the means ± SEM. Significant differences indicated as # *p* < 0.001 vs. blank group, * *p* < 0.05, ** *p* < 0.01, *** *p* < 0.001, and **** *p* < 0.0001 vs. control group were analyzed by one-way ANOVA with Tukey’s post hoc test.

To confirm the inhibitory effect of CES on H_2_O_2_-promoted inducible nitric oxide synthase (iNOS) and ROS expression, we examined intracellular ROS accumulation in a DCFDA flow cytometric assay (Figure 2A). DCFDA is a cell-permeable fluorogenic probe used as an indicator of cellular ROS [31]. After H_2_O_2_ treatment, the ROS level was significantly increased by 29.1% for about 10,000 cells when compared with the blank group, leading to serious oxidative damage in the cells. In contrast, elevated ROS levels due to the induction of H_2_O_2_ were significantly reduced by CES application in a dose-dependent manner, reaching minimum at 8.5% (Figure 2B). We also analyzed cellular iNOS generation by immunocytochemistry. iNOS is a representative key mediator of the oxidative response [32]. The iNOS intensity was significantly increased by H_2_O_2_ treatment in cortical neurons compared with the blank group, whereas CES dose-dependently attenuated H_2_O_2_-induced iNOS production (Figure 2C,E). Furthermore, we investigated whether nuclear factor erythroid 2-related factor 2 (*Nrf2*) signaling is activated following CES treatment under H_2_O_2_-induced oxidative stress using real-time PCR. *Nrf2* is a well-known regulator of anti-oxidative responses and ROS detoxification [33]. The expression of *Nrf2* was significantly reduced by exposure to H_2_O_2_, whereas CES triggered a significant dose-dependent increase of *Nrf2* in H_2_O_2_-treated neurons (Figure 2D). In addition, the Nrf2 protein expression was detected immunocytochemically in each group. The intensity of Nrf2 was higher in the CES groups than in the control group. Significant differences were found between the two different CES (50 and 200 µg/mL) groups and the control group (Supplementary materials, Figure S1). Thus, CES exhibits anti-oxidative neuroprotection properties against H_2_O_2_-induced oxidative stress in cortical neurons.

### 3.3. CES Not Only Promotes Re-Elongation of H_2_O_2_-Injured Axons, but also Accelerates Regenerative Axon Growth of Mature Cortical Neurons after Laceration Injury

**Figure 3:**
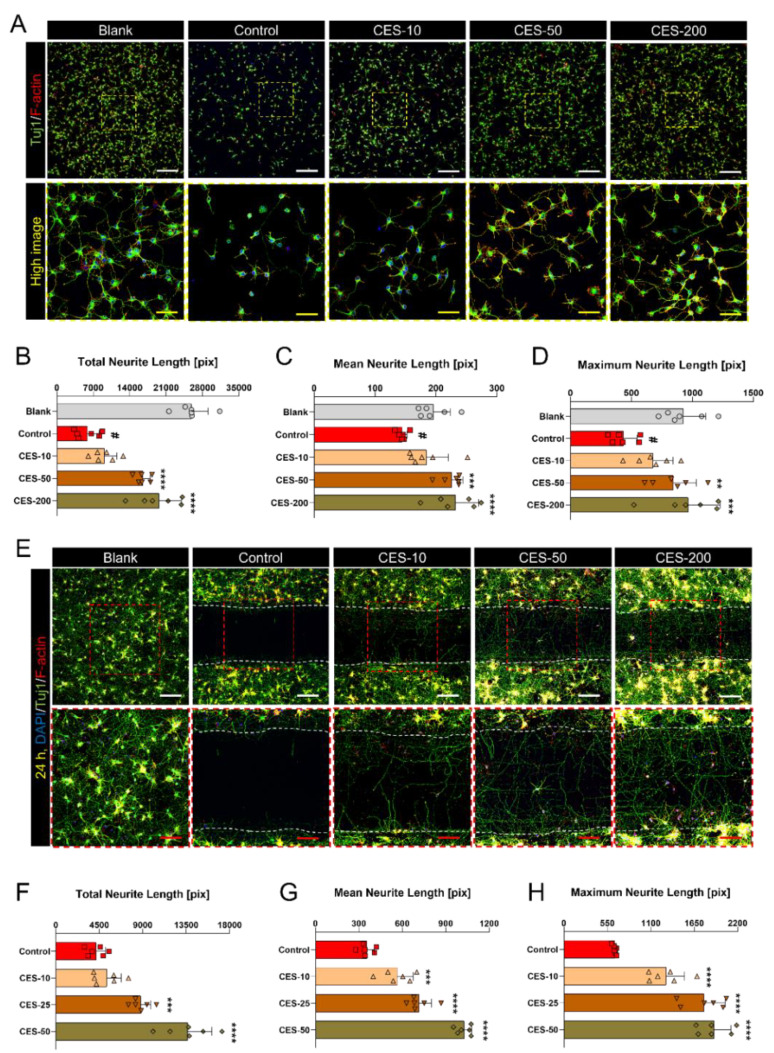
Effect of CES on the promotion of neurite outgrowth and axon regeneration after H_2_O_2_ or laceration-induced injury in cortical neurons. (**A**) Representative image of Tuj1 (green) and F-actin (red) double-staining in H_2_O_2_-treated condition. White scale bar = 200 µM, yellow scale bar = 50 µM. (**B**–**D**) Quantitative analysis of the total, mean, and maximum neurite length in H_2_O_2_-treated condition. (**E**) Representative image of the Tuj1 (green) and F-actin (red) double-staining after laceration injury. White scale bar = 50 µM, red scale bar = 25 µM. (**F**–**H**) Quantitative analysis of the total, mean and maximum neurite length within the lacerated area. Data are expressed as the means ± SEM. Significant differences indicated as # *p* < 0.001 vs. blank group, ** *p* < 0.01, *** *p* < 0.001, and **** *p* < 0.0001 vs. control group were analyzed by one-way ANOVA with Tukey’s post hoc test.

We next evaluated axon regeneration following cortical neuron injury induced by H_2_O_2_ or laceration injury to determine whether CES affects the subsequent axon extension. When H_2_O_2_ was applied to the cortical neurons, the cell populations dramatically declined, leading to cell disconnections. CES effectively stimulated the regrowth in injured axons following H_2_O_2_ induction (Figure 3A). We quantified axonal growth by evaluating three parameters: the total, mean, and maximum neurite length. The results showed that these values were significantly decreased in H_2_O_2_-treated cortical neurons compared with in blank neurons. When neurons were additionally exposed to three doses of CES, these parameters were dose-dependently affected by CES and significantly increased following treatment with 50 and 200 µg/mL CES (Figure 3B–D). Unlike oxidative injury from H_2_O_2_, laceration injury can be mimicked as in vitro traumatic injury and utilized for more intuitive observation of axon regeneration. We therefore applied CES after laceration injury to monitor the acerbating effect on axon regeneration. First, cortical neurons were cultured for 6 days in vitro and were additionally maintained for 1 day after laceration injury and CES treatment. Interestingly, our findings revealed accelerated outgrowth of regenerating axons across the laceration area after CES treatment (Figure 3E). We also examined the difference in neurite growth compared with the control by measuring the total, mean, and maximum neurite length within the laceration area. The length was significantly increased after CES treatment in a dose-dependent manner (Figure 3F–H).

### 3.4. CES Inhibits Conversion of Growth Cone into a Retraction Bulb and Stabilizes Formation of F-Actin Rich Structures in Growth Cone of H_2_O_2_-Treated Cortical Neurons

**Figure 4:**
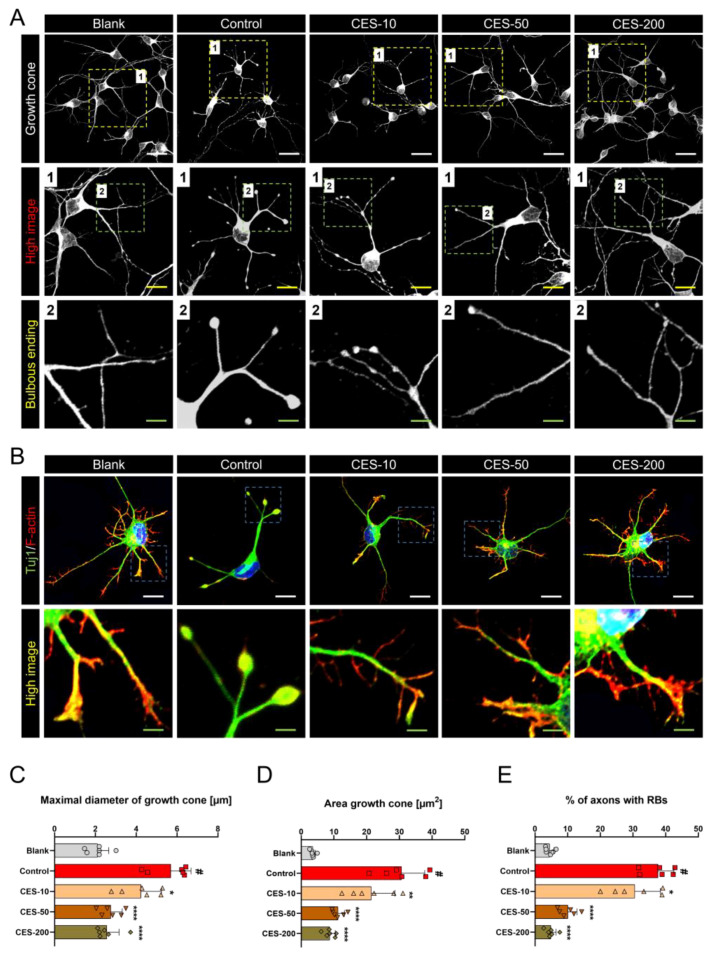
Effect of CES on growth cone F-actin dynamics and morphology in H_2_O_2_-treated cortical neurons. (**A**) Representative images of growth cone in each group. White scale bar = 30 µm, yellow scale bar = 10 µm, green scale bar = 5 µm. (**B**) Representative image of Tuj1 (green) and F-actin (red) double staining. White scale bar = 15 µm, green scale bar = 5 µm. (**C**,**D**) Quantitative analysis of the maximal diameter and area of the growth cone in each group. (**E**) Quantification of the relative percentage of the axons with retraction bulbs (RBs) in H_2_O_2_-treated cortical neurons. Data are expressed as the means ± SEM. Significant differences indicated as # *p* < 0.001 vs. blank group, * *p* < 0.05, ** *p* < 0.01, and **** *p* < 0.0001 vs. control group were analyzed by one-way ANOVA with Tukey’s post hoc test.

Unlike central axons, peripheral axons can regrow after nerve injury. One of the leading causes of axonal regrowth is that the tips of the lesioned axonal stumps in the PNS assemble into new actin-rich growth cones with a stereotypic shape that for allows sustained growth. In contrast, lesioned CNS axons form retraction bulbs at the terminal stumps, which are an oval shape and lack a regenerative drive with axonal swellings and disconnection [34]. Therefore, we focused on morphological changes in the growth cone with a retraction bulb or stereotypic shape, and the F-actin content in the growth cones. Previous studies showed that F-actin plays an important role in guiding microtubule growth during growth cone−target interactions [25]. We found that growth cone collapse and retraction bulbs with F-actin expression formed after H_2_O_2_ treatment. In contrast, CES inhibited conversion of the growth cone into a retraction bulb and stabilized the formation of F-actin-rich filopodium structures in the growth cones of H_2_O_2_-treated neurons (Figure 4A,B). We quantified the change of the growth cone by evaluating three parameters: the maximal diameter and area of the growth cone, and the percentage of axons with a retraction bulb. The maximal diameter of the growth cones was significantly increased after H_2_O_2_ treatment, while treatment with CES significantly decreased the growth cone’s diameter in a dose-dependent manner (Figure 4C). The area of growth cone was further analyzed. The quantification revealed that the relatively low area of growth cone observed in the CES groups was dose-dependent (Figure 4D). All doses of CES provided significantly less formation of the retraction bulb that the control. In addition, we compared the percentage of axons with retraction bulb following CES treatment in H_2_O_2_-injured neurons. A similar trend was observed for the percentage of axons with retraction bulb. Treatment with CES induced significant dose-dependent decreases in the percentage of retraction bulb at the tip of axons (Figure 4E). Our results demonstrate that CES inhibits the generation of retraction bulb from a growth cone in H_2_O_2_-injured neurons, as judged by the morphological criteria.

### 3.5. CES Promotes Re-Elongation of H_2_O_2_-Injured Axons by Enhancing Brain-Derived Neurotrophic Factor and Nerve Growth Factor Expression in Cortical Neurons

**Figure 5:**
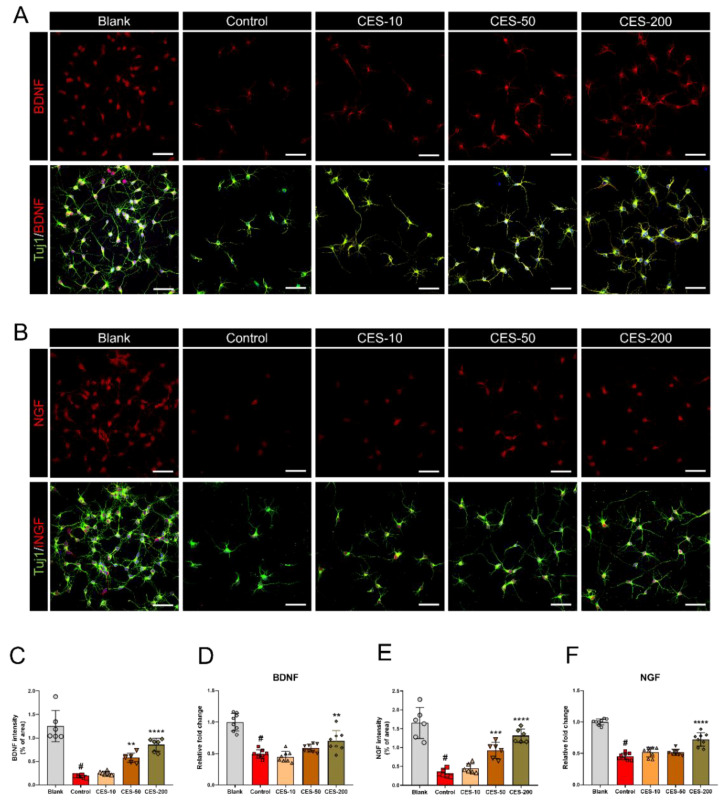
Effect of CES on the BDNF and NGF expression in H_2_O_2_-treated cortical neurons. (**A**,**B**) Representative images of Tuj1 (green) with anti-BDNF (**A**) or NGF (**B**) staining. White scale bar = 50 µm. (**C**,**D**) Quantifying BDNF intensities and mRNA expression level. (**E**,**F**) Quantifying NGF intensities and mRNA expression level. Data are expressed as the means ± SEM. Significant differences indicated as # *p* < 0.001 vs. blank group, ** *p* < 0.01, *** *p* < 0.001, and **** *p* < 0.0001 vs. control group were analyzed by one-way ANOVA with Tukey’s post hoc test.

We also examined BDNF and NGF expression to understand how CES can boost axon regeneration in H_2_O_2_-injured axons. BDNF and NGF can act both locally and systemically, promoting biological repair and axon regeneration, and enhancing synaptic interactions and tissue regeneration [35]. Confocal images revealed that BDNF expression was decreased after H_2_O_2_ treatment, but was preserved well in the CES groups (Figure 5A). Analysis of the relative BDNF intensity showed that the intensity was significantly higher in the 50 and 200 µg/mL CES groups than in the control group (Figure 5C). In addition, *BDNF* mRNA quantification by real-time PCR revealed significant upregulation only in the 200 µg/mL CES group compared with in the control group (Figure 5D). The confocal images of NGF showed a similar trend as that observed in the BDNF images (Figure 5B). CES induced dose-dependent increases in NGF intensity, with significant increases in the 50 and 200 µg/mL CES groups (Figure 5E). The mRNA expression of *NGF* was only significantly upregulated in the 200 µg/mL CES group compared with in the control group (Figure 5F).

### 3.6. CES Attenuates H_2_O_2_-Induced Reduction of Synapsin1 in Cortical Neurons

**Figure 6:**
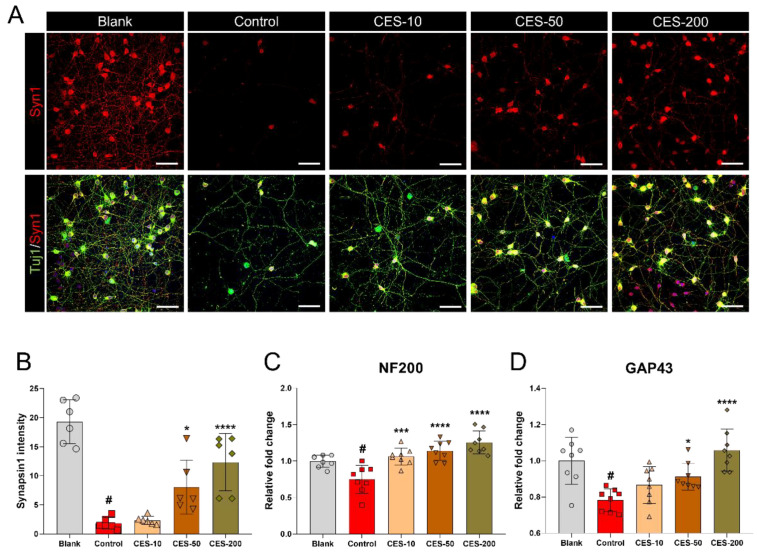
Effects of CES on the altered expression of the synaptic vesicle protein synapsin1 in H_2_O_2_-treated cortical neurons. (**A**) Representative image of Tuj1 (green) and synapsin1 (red) double-staining in H_2_O_2_-treated condition. White scale bar = 50 µm. (**B**) Quantitative analysis of synapsin1 intensity. (**C**,**D**) Quantifying NF200 and GAP43 mRNA expression level. Data are expressed as the means ± SEM. Significant differences indicated as # *p* < 0.001 vs. blank group, * *p* < 0.05, *** *p* < 0.001, and **** *p* < 0.0001 vs. control group were analyzed by one-way ANOVA with Tukey’s post hoc test.

Finally, to determine whether CES can induce synapse formation in H_2_O_2_-injured neurons, synapsin1 staining was performed as an indirect marker of synapse formation [36]. Synapsin1 normally appeared as bright signals within the cell soma and axons, whereas the synapsin1 signal was decreased following the addition of H_2_O_2_. In contrast, treatment with CES significantly and dose-dependently increased the synapsin1 signal (Figure 6A). The synapsin1 intensity significantly differed between the 50 and 200 µg/mL CES and control groups, with a mean of 3–6-fold improvement (Figure 6B). Furthermore, we confirmed the effect of CES on the expression of regeneration-associated genes including neurofilament 200-kDa (*NF200*) and growth-associated protein (*GAP43*). CES induced a dose-dependent increase in *NF200* expression, with a significant difference between all CES doses and the control group (Figure 6C). The expression level of *GAP43* was also increased after CES treatment in a dose-dependent manner, with a significant difference between the 50 and 200 µg/mL CES groups and control group (Figure 6D).

## 4. Discussion

One of the major challenges in treating neurological disorders is ameliorating the hostile environment after nerve injury. Environmental stress is caused by ROS generation and accumulation, which can lead to oxidative damage in cells. Under normal physiological conditions, cellular homeostasis is achieved by the fine redox balance between ROS production and scavenging. However, this ROS production-scavenging system does not function normally after injury, and cells suffer from oxidative damage [37]. Therefore, therapeutic strategies targeting oxidative stress may enable neurological recovery in patients. Numerous antioxidative substances have been identified in animal, plants, food, bacteria, fungi, and algae, and have been investigated to determine their antioxidant effects [38]. Particularly, have gained attention in recent years because of their low risk of adverse effects, and they have been empirically verified to be safe and effective [39].

CES has traditionally been used to treat muscle atrophy, weakness, chronic infectious symptoms, fractures, and joint weakness. Furthermore, CES was shown to possess pharmacological activities and has been utilized to develop health/functional food products. However, there are safety concerns regarding the appropriate dose of deer velvet that does not cause adverse effects. Additionally, the appropriate therapeutic doses range of deer velvet for treating neurological diseases has not been determined. A study by Xin et al. showed that sika deer (*Cervus nippon* Temminck) velvet antler polypeptides prevented SH-SY5Y cell death by affecting the phosphorylated c-Jun N-terminal kinase pathway via caspase-12-mediated apoptosis, and the viability of SH-SY5Y cells was significantly increased by 125, 250, and 500 μg/mL velvet antler polypeptides [19]; however, the authors did not directly demonstrate the relevance of these effects in neurons.

Here, we demonstrated that up to 500 µg/mL CES did not cause neurotoxicity to primary cortical neurons, and the optimal therapeutic dose range of CES was determined as 10–500 µg/mL. We also found that CES was highly effective for promoting neuronal survival and neurite outgrowth by inhibiting oxidation and necrosis under H_2_O_2_-treated conditions. Axon regeneration was observed following CES treatment after laceration injury, with an increase in both neurite outgrowth and axon regeneration in the lacerated area. This mechanical laceration injury is the preferred in vitro model for studying neuronal injuries induced by spinal cord injury or traumatic brain injury [29]. Therefore, CES after injury in cortical neurons obviously and robustly facilitated axon regeneration. Although we did not verify the active components of deer antler that exert a neurotherapeutic effect, the neuronal activity of deer antler is thought to have originated in the gangliosides [40]. Previous studies have revealed that gangliosides contribute significantly to support the formation and stabilization of functional synapses, neural circuits, axonal growth, and neuronal differentiation [41].

This successful regeneration process must be preceded by the successful production of new growth cones. Interestingly, H_2_O_2_ treatment leads to growth cone collapse and to the formation of a characteristic swelling end-bulb structure. Cultured cortical neurons also showed reduced F-actin-expressing growth cones on the ends of growing axons at 24 h after H_2_O_2_ treatment. Meanwhile, new growth cones were more abundant after the cells were treated with CES. We also found that the expression of neurotrophic factors (BDNF and NGF) and regeneration-associated genes (*NF200*, *GAP43,* and *Nrf2*) was enhanced by CES treatment. Once regeneration-associated gene expression is stopped by injury, it may not be re-induced to promote regeneration. However, CES may stimulate the growth factors and concomitantly induce axon regeneration from oxidative damage. Although CES-induced alteration may promote growth cone reformation and axon regeneration, the mechanism underlying growth cone reorganization induced by CES remains unclear. A previous study reported that doublecortin-like kinases, known as doublecortin and CaM kinase-like, are essential growth cone reformation-associated proteins. In addition, doublecortin members regulate F-actin dynamics in injured axonal stumps [42]. Another limitation of this study was that we only evaluated the in vitro effect of CES on the neurotherapeutic potential. Thus, it is difficult to interpret functional recovery at the tissue level. Further animal studies are needed to clarify the therapeutic effect of CES. Additionally, the composition, mechanical properties, and structure of deer antler bone must be examined. Deer antler bone is divided into the beam, crown, trez, bez, and brow tines according to its branching structure [43,44]. Although each portion is thought to have different effects, studies focused on these neurological aspects have not been performed. Comparing the effects of different parts of deer antler may provide insight into the underlying mechanisms, facilitating the development of more effective and specialized therapeutic strategies.

## 5. Conclusions

CES applied to cortical neurons may improve axon regeneration and growth cone reformation following H_2_O_2_-induced oxidative injury.

## Figures and Tables

**Table 1:** Primer sequences used for the real-time PCR analysis.

Gene	5′-3′	Primer Sequence
*Nrf2*	Forward	GATCTGTCAGCTACTCCCAG
	Reverse	GCAAGCGACTCATGGTCATC
*BDNF*	Forward	CTTGGAGAAGGAAACCGCCT
	Reverse	GTCCACACAAAGCTCTCGGA
*NGF*	Forward	CCAAGGACGCAGCTTTCTATC
	Reverse	CTGTGTCAAGGGAATGCTGAAG
*NF200*	Forward	AACACCACTTAGATGGCGGG
	Reverse	ACGTGGAGCGTTCAGCAATA
*GAP43*	Forward	TGCCCTTTCTCAGATCCACT
	Reverse	TTGCCACACAGAGAGAGAGG
*GAPDH*	Forward	CCCCCAATGTATCCGTTGTG
	Reverse	TAGCCCAGGATGCCCTTTAGT

## Data Availability

The data presented in this study are available upon request from the corresponding author.

## Supplementary Materials

The following are available online at https://www.mdpi.com/article/10.3390/biology10090833/s1. Figure S1: Nrf2 immunocytochemical analysis of cortical neuron treated with 10, 50, and 200 µg/mL of CES with H_2_O_2_ exposure.
