# Supplementary material for: Neurotherapeutic Potential of Cervus elaphus Sibericus on Axon Regeneration and Growth Cone Reformation after H2O2-Induced Injury in Rat Primary Cortical Neurons
Source: Biology (Basel). 2021 Aug 26;10(9):833. doi: 10.3390/biology10090833 (PMC8471249; doi:10.3390/biology10090833)
Supplement: Supplementary file 1 [file biology-10-00833-s001.zip › biology-1347148-supplementary.pdf]

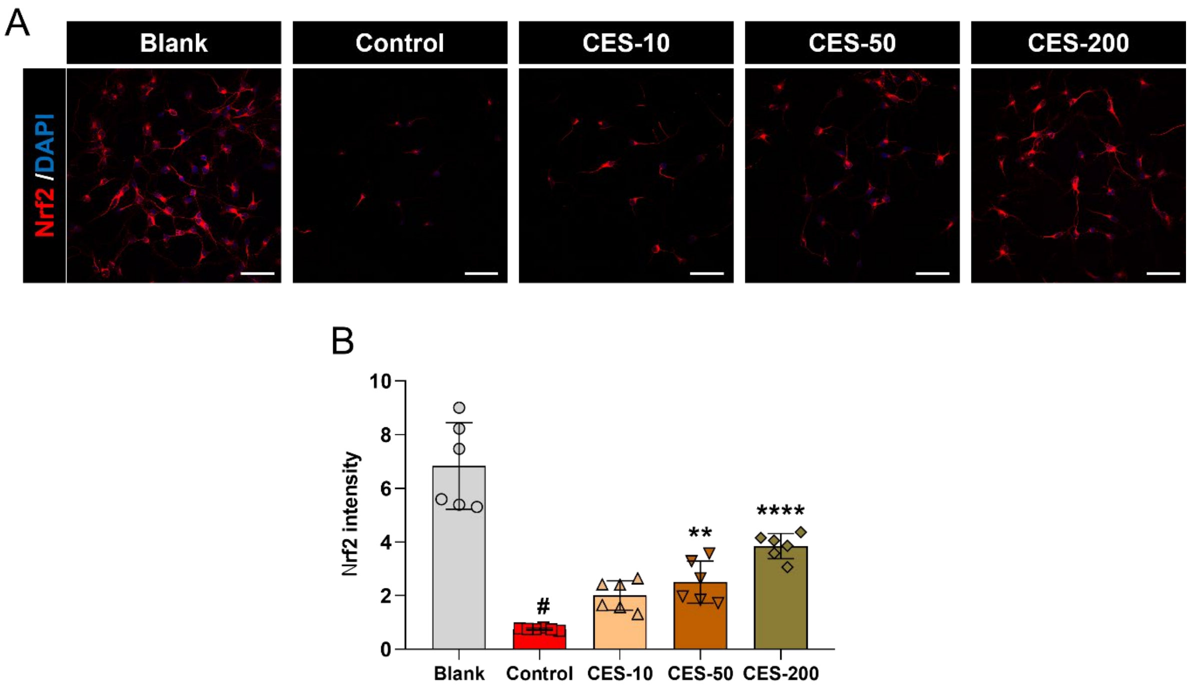

Supplementary FigureS1. Nrf2 immunocytochemical analysis of cortical neuron treated with 10, 50 and 200 µg/ml of CES with H_2O_2 exposure. (A) Representative immunocytochemical images showing Nrf2 (red) in H_2O_2-treated cortical neurons with 10, 50 and 200 µg/ml of CES. White scale bar = 50 µm. (B) Quantification of Nrf2 intensity in each group. Data represent mean ± SEM of six independent experiments. Significant differences indicated as $^{\#}p < 0.001$ compared vs. the blank group, $^{**}p < 0.01$ and $^{****}p < 0.0001$ vs. the control group were analyzed via one-way ANOVA with Tukey's post-hoc test.
